# RGD-Functionalized Hydrogel Supports the Chondrogenic Commitment of Adipose Mesenchymal Stromal Cells

**DOI:** 10.3390/gels8060382

**Published:** 2022-06-15

**Authors:** Cristina Manferdini, Diego Trucco, Yasmin Saleh, Elena Gabusi, Paolo Dolzani, Enrico Lenzi, Lorenzo Vannozzi, Leonardo Ricotti, Gina Lisignoli

**Affiliations:** 1 IRCCS Istituto Ortopedico Rizzoli, SC Laboratorio di Immunoreumatologia e Rigenerazione Tissutale, 40136 Bologna, Italy; cristina.manferdini@ior.it (C.M.); diego.trucco@santannapisa.it (D.T.); yasmin.saleh@ior.it (Y.S.); elena.gabusi@ior.it (E.G.); paolo.dolzani@ior.it (P.D.); enrico.lenzi@ior.it (E.L.); 2 The BioRobotics Institute, Scuola Superiore Sant’Anna, 56025 Pisa, Italy; lorenzo.vannozzi@santannapisa.it (L.V.); leonardo.ricotti@santannapisa.it (L.R.); 3 Department of Excellence in Robotics & AI, Scuola Superiore Sant’Anna, 56025 Pisa, Italy

**Keywords:** adipose mesenchymal stromal cells, hydrogels, chondrogenic differentiation, RGD motif, hydrogel characterization, cartilage regeneration

## Abstract

Articular cartilage is known to have limited intrinsic self-healing capacity when a defect or a degeneration process occurs. Hydrogels represent promising biomaterials for cell encapsulation and injection in cartilage defects by creating an environment that mimics the cartilage extracellular matrix. The aim of this study is the analysis of two different concentrations (1:1 and 1:2) of VitroGel^®^ (VG) hydrogels without (VG-3D) and with arginine-glycine-aspartic acid (RGD) motifs, (VG-RGD), verifying their ability to support chondrogenic differentiation of encapsulated human adipose mesenchymal stromal cells (hASCs). We analyzed the hydrogel properties in terms of rheometric measurements, cell viability, cytotoxicity, and the expression of chondrogenic markers using gene expression, histology, and immunohistochemical tests. We highlighted a shear-thinning behavior of both hydrogels, which showed good injectability. We demonstrated a good morphology and high viability of hASCs in both hydrogels. VG-RGD 1:2 hydrogels were the most effective, both at the gene and protein levels, to support the expression of the typical chondrogenic markers, including collagen type 2, SOX9, aggrecan, glycosaminoglycan, and cartilage oligomeric matrix protein and to decrease the proliferation marker MKI67 and the fibrotic marker collagen type 1. This study demonstrated that both hydrogels, at different concentrations, and the presence of RGD motifs, significantly contributed to the chondrogenic commitment of the laden hASCs.

## 1. Introduction

The degeneration of articular cartilage due to trauma, osteoarthritis, or aging is a common joint disorder, with a high incidence worldwide [1,2]. It has been reported that the articular cartilage has limited intrinsic self-healing capacity, mainly due to its avascular and aneural nature [3]. Different clinical treatments, such as autologous chondrocyte implantation, mosaicplasty, and microfracture, have been used for inducing articular cartilage regeneration [4]. However, these techniques show limitations since they do not permit effective long-term cartilage regeneration and do not assure the formation of a fully differentiated articular cartilage structure, thus requiring the development of alternative strategies [5].

Tissue engineering approaches represent a promising alternative for cartilage regeneration and repair [6,7,8]. Hydrogels are among the most versatile kinds of materials used for various tissue engineering applications, since they can be engineered into almost any shape and size [9,10]. They can also be functionalized or enriched with micro/nanofillers for building composite hydrogels, giving them improved properties tailored to specific applications [11,12]. In general, hydrogels represent hydrophilic 3D networks composed of water-soluble natural (e.g., polysaccharides and proteins) and/or synthetic polymers crosslinked by chemical or physical methods to form a water-insoluble matrix [13,14]. Different from hydrogels, aerogels are porous materials obtained when the liquid phase of a hydrogel is replaced by a gas, while preserving the internal structure and the surface area of the initial hydrogel [15,16,17]. They have been proven to provide highly desirable 3D environments for the regeneration of the cartilaginous tissue, as shown by in vitro and in vivo studies [18,19,20,21]. Hydrogels are viscoelastic materials, and their mechanical properties are an important requirement for engineering cell functions, since their tuning can improve mechanosensitive signaling. In fact, matrix stiffness, permeability, swelling ability, and degradation provide a peculiar biomimetic environment able to create a niche suitable to drive cell migration, adhesion, proliferation, and chondrogenic differentiation [22]. These properties, associated with other factors (i.e., seeding conditions, hypoxia), modulate the differentiation processes [23,24,25].

The use of injectable and in situ-forming hydrogels enables the treatment of irregular cartilage defects and a proper alignment with the surrounding tissues, characteristics that make them superior to 3D-structured scaffold-based approaches [26,27]. Meanwhile, from the clinical viewpoint, implantation surgery can be avoided and replaced by a simple, minimally invasive injection [26,28]. Moreover, bioactive molecules or cultured cells can simply be incorporated into the hydrogel precursors before they are ready for injection, or 3D bioprinted [29,30,31].

Mesenchymal stromal cells (MSCs) have shown interesting results in the field of cartilage regeneration, mainly due to their accessibility, immunomodulatory and pro-regenerative capabilities, and chondrogenic differentiation potential [32,33,34,35]. It has been shown that MSCs from various origins, combined with scaffold materials, have great potential in the regeneration of cartilage, in both animal models and in humans, as suggested by recent clinical trials [27,28,30,36]. It has been recognized that MSCs, laden in natural or synthetic hydrogels, create a suitable environment for inducing their cellular differentiation [37].

Recently, it has been shown that VitroGel^®^ (VG) hydrogels have the potential to mimic the cartilage extracellular matrix (ECM) [38]. This hydrogel can also provide binding sites for cell adhesion, thanks to the functionalization with arginine-glycine-aspartic acid (RGD) motifs, which is a well-known tri-peptide able to promote cell attachment and, at the same time, favor cell-matrix interactions by enhancing cellular function, like cell proliferation, migration, and differentiation [39,40]. It has been shown that the RGD motif is a crucial component of adhesive proteins in the ECM, working through integrin transmembrane receptors by transmitting the cell survival signaling within the cells [41]. However, it has been shown that this tri-peptide exerts controversial issues in cartilage tissue engineering [42]. It has been demonstrated that the hydrogel modified with RGD peptide (VG-RGD) can be easily injected for the treatment of the intervertebral disc in rats, promoting the proliferation and differentiation of nucleus pulposus (NP)-derived MSCs (NPMSCs), and also promoting the NPMSC’s long-term retention and survival in the degenerated intervertebral disc, with the formation of a neo-ECM [38].

The chondrogenic commitment of human adipose-derived mesenchymal stromal cells (hASCs) in VG hydrogels has been never investigated before. The novelty of the study is to gain new insight into the microenvironment fostered by natural hydrogels, by investigating the 3D hydrogel environment created by VG without (VG-3D) and with RGD (VG-RGD) motif on the chondrogenic differentiation of encapsulated hASCs for the potential treatment of cartilage defects.

## 2. Materials and Methods

### 2.1. Materials

VG-3D and VG-RGD hydrogels were both purchased from Well Bioscience (North Brunswick, NJ, USA) and prepared following the manufacturer’s protocol. Briefly, the VG-3D and VG-RGD solutions were directly mixed at room temperature (RT) with the Dilution Solution Type 1^®^ (The Well Bioscience, North Brunswick, NJ, USA) at the ratio of 1:1 and 1:2 to obtain two different hydrogel formulation. Then, Dulbecco Modified Eagle Medium (DMEM) (Life Technologies, Bleiswijk, The Netherlands) was added to each pre-crosslinked solution at the ratio of 4:1 (pre-crosslinked solution: DMEM) at RT. Four different combinations (VG-3D 1:1, VG-3D 1:2, VG-RGD 1:1, VG-RGD 1:2) were analyzed. Sodium alginate powder (Sigma-Aldrich Merck, Saint Louis, MO, USA) was used as the control hydrogel.

### 2.2. Experimental Plan

**Figure 1:**
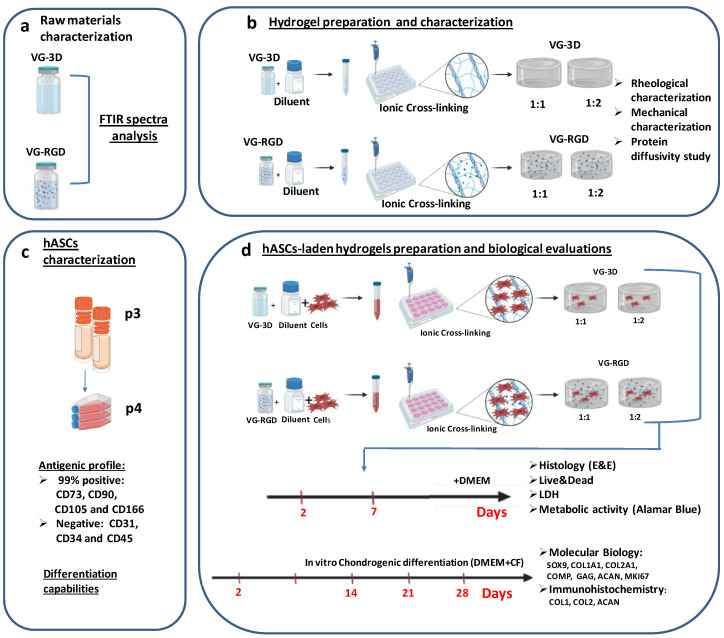
Depiction of the experimental plan: (**a**) characterization of raw materials through FTIR analysis; (**b**) evaluation of the rheological and mechanical properties of hydrogels and protein diffusivity evaluation; (**c**) characterization of the hASCs in terms of antigenic profile and differentiation capabilities; (**d**) biological analysis to assess cell distribution, viability, cytotoxicity and metabolic activity, and chondrogenic assessment evaluated through molecular biology assays and immunohistochemical staining.

The experimental workflow for this study is shown in Figure 1. Briefly, the VG hydrogels raw materials (Figure 1a) were characterized through FTIR analysis. Then, the rheological and mechanical properties of hydrogels (Figure 1b) were evaluated and the protein diffusivity was tested. hASCs were analyzed in terms of antigenic profile and differentiation capabilities (Figure 1c). Then, hASC-laden hydrogels (Figure 1d) were analyzed at two different time points to assess cell distribution, viability, cytotoxicity, and metabolic activity. Finally, the chondrogenic differentiation of hASCs encapsulated in hydrogels (Figure 1d) was evaluated through molecular biology assays and immunohistochemical staining.

### 2.3. Raw Materials Characterization

#### 2.3.1. FTIR Analysis

Fourier Transform Infrared Spectroscopy (FTIR) was used to characterize the presence of specific chemical groups in the VG-3D and VG-RGD hydrogels (Figure 1a). VG-3D and VG-RGD (300 µL) solutions were dehydrated using a freeze dryer (Labconco, FreeZone 2.5 Plus, Kansas City, MO, USA) for two days. VG-based material powders were analyzed and compared with sodium alginate powder. All FTIR spectra were obtained in the wavenumber range from 4000 to 600 cm^−1^ during 64 scans, with 4 cm^−1^ resolutions (Shimadzu IRAffinity-1, Duisburg, Germany). The FTIR spectra were plotted using OriginPro 8.5 (OriginLab, Northampton, MA, USA).

#### 2.3.2. Hydrogel Preparation and Characterization

##### Rheological Characterization

The rheological properties of VG-3D and VG-RGD hydrogels were measured at 25 °C using a rheometer (Anton Paar MCR-302, Graz, Austria) with a plate-plate geometry (diameter: 25 mm, distance gap: 1 mm). The flow behavior was measured on all blends immediately after mixing with DMEM for shear rates ranging from 0.1 to 1000 s^−1^ in rate-controlled mode by selecting 10 points for each decade. The viscosity was investigated to model the viscosity of the materials according to the following power law [43]:

(1) $$\eta =K{\dot{\gamma }}^{n-1}$$

where **η** is the dynamic viscosity, $\dot{\gamma }$ is the fluid shear rate, and **K** and **n** are the consistency index and the flow behavior index, respectively. Through linear interpolation, **K** and **n** for both VG-3D and VG-RGD hydrogels were determined and used to model the shear stress applied to cells during the injection (e.g., using a 26 G needle) following the model for non-Newtonian fluids [44].

##### Mechanical Characterization

To assess mechanical properties, 300 µL of VG-3D and VG-RGD solutions were gently poured into polydimethylsiloxane (PDMS) molds (diameter: 10 mm, height: 3 mm). After 20 min of stabilization at RT to let the solution crosslink, a further 200 µL of DMEM was placed over the hydrogels. Then, samples were incubated overnight at 37 °C and 5% CO_2_. The mechanical characterization was performed the day after the samples’ preparation on all four groups of crosslinked hydrogels (n = 6/group). The elastic modulus was measured by performing uniaxial compression with an Instron Mechanical Testing System (model 2444, Instron, Norwood, MA, USA) equipped with ±10 N load cell at a compression rate of 1 mm/min until reaching the hydrogel breaking point, as previously reported [45].

##### Protein Diffusivity Study

Albumin from bovine serum (BSA) conjugated with FITC (Invitrogen, FITC-BSA, Carlsbad, CA, USA) was tested to investigate the protein diffusion in VG-3D and VG-RGD hydrogels. FITC-BSA solution (1 mL, concentration: 100 µg/mL) was poured onto the upper surface of each crosslinked hydrogel. After 30 min, the hydrogels were washed with phosphate buffer saline (PBS 1x, Corning) 3 times for 5 min at 37 °C to remove the residual fluorescent dye. Controls (CTR-3D and CTR-RGD) without FITC-BSA were also used to verify the hydrogel autofluorescence. The cross-sectioned surface was observed with a confocal laser scanning microscope (Nikon Eclipse Ti, Tokyo, Japan) at the FITC wavelength (488 nm).

### 2.4. hASCs Characterization

#### 2.4.1. Cell Culture and Phenotypic Characterization of hASCs

hASCs were purchased from Lonza (Morrisville, NC, USA (n = 5) and were expanded by seeding 7500 cells/cm^2^ in T150 culture flask and culturing in α-MEM containing 5% isogrowth (IsoCellsGROWTH, Euroclone, Pero, Italy) and 1% penicillin/streptomycin (Life Technologies) at 37 °C in 5% CO_2_ incubator_._ Before hydrogel encapsulation (Figure 1c), hASCs were phenotypically characterized for the CD markers CD31, CD34, CD45, CD73, CD90, CD105, and CD166, as we previously reported [46], and were analyzed for differentiation capabilities by using specific osteogenic and chondrogenic mediums, as we previously described [34,46] to check that they satisfied the “minimal criteria for defining multipotent MSCs,” as previously reported [47].

#### 2.4.2. hASCs-Laden Hydrogel Preparation and Biological Evaluations

VG-3D and VG-RGD hydrogels were prepared according to the VitroGel^®^ protocol guidelines indicated by the company and following a defined workflow (Figure 1d). Briefly, both hydrogels were first diluted with the Dilution Solution Type 1^®^ (1:1 and 1:2) at RT. Then, the hydrogel dilution was mixed with the ASCs (2 × 10^6^ cell/mL) at a 4:1 ratio. Next, 300 µL of the mixture was gently transferred into the cell crown (Scaffdex, Finland), and inserted into the 24-well plates. After 20 min of stabilization at RT, a further 200 µL of DMEM was placed over the hydrogel.

Before testing the chondrogenic differentiation, hydrogels-loaded hASCs were cultured for 2 and 7 days in DMEM and evaluated for cell distribution, cell viability, cytotoxicity, and metabolic activity.

#### 2.4.3. Histology

On days 2 and 7, VG-3D and VG-RGD hydrogel-loaded ASC were fixed in 10% formaldehyde in DPBS for 40 min, washed in DPBS, dehydrated in ethanol, and embedded in paraffin. Sections of 5 µm were cut and stained with hematoxylin-eosin (Bioptica, Milan, Italy), and slides were analyzed using a light microscope, according to the manufacturer instructions.

#### 2.4.4. Live/Dead Viability Assay

The viability of hASC encapsulated in VG-3D and VG-RGD was evaluated using the Live/Dead kit (Life Technologies). Hydrogels were washed in D-PBS (Aurogene Srl, Rome, Italy) and incubated with Live/Dead solution for 35 min at 37 °C. After staining, hydrogels were washed again with DPBS and evaluated, with living cells showing green and the nuclei of dead cells showing red when viewed with a fluorescence microscope (Nikon Instruments Europe BW, Amsterdam, The Netherlands).

#### 2.4.5. LDH Assay

The cytotoxicity of different hydrogel concentrations on hASCs was detected using the LDH assay kit (Roche, Mannheim, Germany). The supernatant was collected after 2 and 7 days and tested for the absorbance values at 490 nm using a microplate reader TECAN Infinite^®^ 200 PRO (Tecan Italia S.r.l., Cernusco Sul Naviglio, Italy).

#### 2.4.6. Metabolic Activity Test

Cell metabolic activity was analyzed at day 2 and 7 using the Alamar blue test. Briefly, the samples were incubated with 10% Alamar blue (Life Technologies), and after 5 h, the absorbance was read at 570 and 600 nm using an automated spectrophotometric plate reader TECAN Infinite^®^ 200 PRO (Tecan). The results were expressed as percentages of AlamarBlue reduction, as indicated by the manufacturer’s data sheet (BioRad Laboratories, Hercules, CA, USA).

#### 2.4.7. Chondrogenic Differentiation

hASCs laden both in VG-3D and VG-RGD (1:1 and 1:2) were treated with chondrogenic medium (high-glucose DMEM supplemented with 50 mg/mL ITS + premix, 10^−7^ M dexamethasone, 50 μg/mL ascorbate–2phosphate, 1-mM sodium pyruvate, and 100 U/mL–100 μg/mL penicillin–streptomycin, Sigma Aldrich, St. Louis, MO, USA) containing chondrogenic factors (+CF) TGF-β3 10 ng/mL and BMP6 10 ng/mL (both from Miltenyi Biotech, Auburn, CA, USA). The cell culture medium was changed three times a week. Each construct was analyzed on day 2, 14, 21, and 28 to test the chondrogenic markers.

#### 2.4.8. Molecular Biology

Total RNA was extracted from all chondrogenic samples at day 2, 14, 21, and 28. Each construct sample was treated with 1 mL of Eurogold RnaPure (EuroClone S.p.a.), immediately snap frozen in liquid nitrogen (−196 °C), and stored in a freezer at −80 °C. RNA extraction was performed by homogenizing samples and following the Eurogold manufacturer’s instruction. The samples were then treated with DNase I (DNA-free Kit) and RNA quantified using a Nanodrop^®^ spectrophotometer (EuroClone S.p.a). Reverse transcription was performed using a Super Script^®^ Vilo™ cDNA synthesis kit (Life Technologies), according to the manufacturer’s protocol. Real-time polymerase chain reaction (PCR) was performed using LightCycler^®^2.0 [48] (Roche Molecular Biochemicals, Mannheim, Germany) for the quantification of the following genes: SRY-Box Transcription Factor 9 (*SOX9)*, collagen type 1 alpha 1 chain (*COL1A1*), collagen type 2 alpha 1 chain (*COL2A1*), cartilage oligomeric matrix protein (*COMP*), glycosaminoglycan (*GAG*), aggrecan (*ACAN*), and the cellular marker of proliferation Ki-67 (*MKI67)* (Table S1). The efficiency of all primers was confirmed to be high (>90%) and comparable (Table S1). For each target gene, mRNA levels were calculated, normalized to the housekeeping gene Glyceraldehyde-3-Phosphate Dehydrogenase (*GAPDH)* according to the formula 2^−ΔCt^, and expressed as a percentage of the reference gene.

#### 2.4.9. Immunohistochemistry Staining

On day 28, both concentrations of VG-3D and VG-RGD hydrogel-loaded ASC were fixed in 10% formaldehyde in DPBS for 40 min, washed in PBS, dehydrated in ethanol, and embedded in paraffin. Immunohistochemistry techniques were used to evaluate collagen type 1, collagen type 2, and aggrecan expression. Serial sections of 5 µm were incubated for 60 min at RT with monoclonal mouse anti-human collagen type 1 (diluted 1:40), anti-human collagen type 2 (diluted 1:20), and anti-human aggrecan (diluted 1:50, all from Chemicon International, Temecula, CA, USA), rinsed, and then sequentially incubated at RT for 20 min with multilinker biotinylated secondary antibody and alkaline phosphatase-conjugated streptavidin (Biocare Medical, Walnut Creek, CA, USA). The colorimetric reactions were developed using fast red (Biocare Medical), counterstained with hematoxylin, and mounted with glycerol jelly. The sections were evaluated with a bright field microscope (Nikon Instruments Europe BW). Negative and isotype matched control sections were tested.

#### 2.4.10. Statistical Analysis

Statistical analysis was done using CSS Statistical Software (Statsoft Inc., Tulsa, OK, USA). Non-parametric tests were performed, since the data did not have normal or strongly asymmetric distribution. The Kruskal–Wallis with Dunn’s multiple comparisons test was used and values of *p* < 0.05 were considered statistically significant. Values were expressed either as the median and minimum and maximum, or as mean ± SD.

## 3. Results and Discussion

### 3.1. Raw Materials Characterization: FTIR Analysis

**Figure 2:**
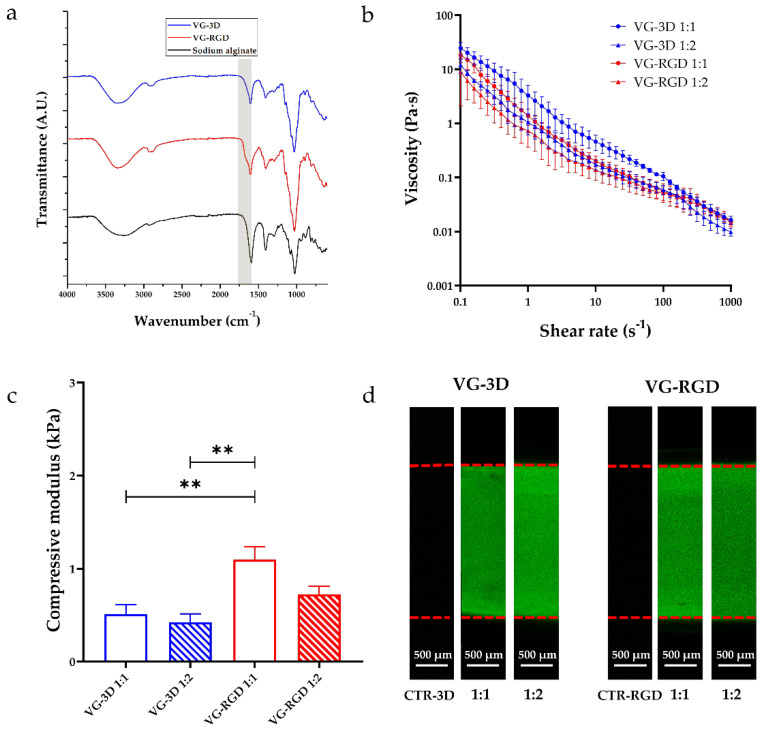
(**a**) FTIR analysis of lyophilized VG-3D (blue), VG-RGD (red), and control sodium alginate powder (black) obtained in the range of wavenumber from 4000 to 600 cm^−1^; (**b**) rheological characterization of VG-3D and VG-RGD materials regarding viscosity (flow curves); (**c**) mechanical characterization of VG-3D and VG-RGD hydrogels in terms of compressive modulus; (**d**) confocal images of cross-sectioned VG-3D (**left**) and VG-RGD (**right**) crosslinked hydrogels (1:1 and 1:2 dilutions) after treatment with FITC-conjugated albumin to evaluate the protein diffusivity through 3D constructs. The controls (CTR-3D, CTR-RGD) represent both hydrogels, not treated with FITC-conjugated albumin. The red dashed lines represent the hydrogel’s thickness. Data are presented as mean ± SD, n = 5; *p*-values are calculated by the Kruskal–Wallis method with Dunn’s multiple comparisons test, as indicated in the statistical section, ** *p* < 0.001.

Firstly, the FTIR spectra analysis of both commercial VG-3D and VG-RGD solutions was performed to identify the functional groups present in these materials. This assessment was also performed on the sodium alginate, which was found to be similar in terms of functional groups, as a comparison. As indicated by the manufacturer, both VG hydrogels are xeno-free synthetic polysaccharide-based hydrogel systems, with or without RGD motif, that can be crosslinked using ions present in DMEM solution. Therefore, we chose to compare alginate, which is a natural polysaccharide that normally crosslinks using magnesium or calcium ions (both present in the datasheet of the DMEM solution), used in many scientific studies related to tissue engineering and regenerative medicine [49]. For these reasons, we used this compound as a control for FTIR analysis to verify and discuss if differences existed in the presence of specific functional groups with a well-known polysaccharide. As shown in Figure 2a, all materials exhibited typical peaks at 3430 cm^−1^, 2916 cm^−1^, 1424 cm^−1^, and 1025 cm^−1^. Moreover, in VG-based materials, we found signal peaks around 1623 cm^−1^ and 1075 cm^−1^, but absent in the sodium alginate. The peak at 3430 cm^−1^, attributed to the O–H stretching vibration, was present in all materials. Interestingly, the peak at 2916 cm^−1^ in VG-based materials, indicative of the stretching vibration of all saturated C–H bonds, was more evident than in sodium alginate. As shown in Figure 2a, the signal, due to the presence of the tripeptide Arg-Gly-Asp (RGD) (peptides peak around 1670 cm^−1^), is also noticeable (yellow band) on the VG-RGD spectrum, which identifies the minimal sequence to recognize the fibronectin that mediates cell attachment [27,28]. In particular, the signal overlapped with the peak around 1670–1623 cm^−1^, which is attributed to the stretching vibration of the COO− groups. Moreover, the second C–N stretching vibration peak at 1385 cm^−1^ was common for all materials, not just for the VG-RGD hydrogel [29]. As for the sodium alginate, the peak at 1424 cm^−1^ for the symmetric stretching of the COO− groups and the peak at 1025 cm^−1^ attributed to the OC–O–CO stretching vibrations were also present in VG-based hydrogels. However, the peak at 1075 cm^−1^ attributed to C–O–C was not found in both VG-based materials, while it was found in sodium alginate’s spectrum [30]. Overall, the FTIR analysis indicates that both VG-3D and VG-RGD formulations feature signal peaks similar to those of sodium alginate, which is known to support the chondrogenic differentiation of MSCs [34].

### 3.2. Hydrogel Preparation and Characterization

#### 3.2.1. Rheological Properties

The rheological properties (Figure 2b) of all solutions, immediately after mixing the components (thus in the pre-crosslinked formulation), were evaluated to analyze the flow curve and the relative shear stress applied to cells during injection. Figure 2b shows the flow curves of VG-3D and VG-RGD at both dilutions tested (1:1 and 1:2). At 1 s^−1^, the viscosity of both VG-based hydrogels showed similar values. In particular, the viscosity of VG-3D 1:1 and 1:2 was 3.29 ± 1.79 Pa·s and 1.60 ± 0.67 Pa·s, respectively, and the viscosity of VG-RGD 1:1 and 1:2 was 1.37 ± 0.45 Pa·s and 1.77 ± 0.76 Pa·s, respectively.

The rheological indexes typical of non-Newtonian fluids (n and K) were extracted by the flow curves shown in Figure 2b, and the corresponding shear stresses (τ) were calculated, hypothesizing the delivery of the hydrogels through a 26 G needle (Table 1).

All VG-based hydrogels showed a shear-thinning behavior (**n** < 1), a relatively low consistency index, and a viscosity that decreased when the shear rate increased [31]. These results suggest that all combinations can be injected (also directly in situ), as previously suggested elsewhere [24]. The VG-3D hydrogels showed a higher consistency index than VG-RGD ones, and we noticed a difference in both VG-based hydrogels while varying the dilution factor from 1:1 to 1:2 (Table 1). The results showed that the shear stress applied to cells encapsulated in all solutions during an injection step through a 26 G needle was lower than the safety threshold equal to 5 kPa, calculated as previously reported [32].

#### 3.2.2. Mechanical Properties

The compressive modulus of all VG-based hydrogels is shown in Figure 2c. After crosslinking, VG-3D (1:1 and 1:2) showed a compressive modulus equal to 0.51 ± 0.10 kPa and 0.41 ± 0.09 kPa, respectively, and no statistical differences were found. VG-RGD 1:1 showed an elastic modulus of 1.10 ± 0.13 kPa, significantly higher than both VG-3D hydrogels (** *p* < 0.01), and VG-RGD 1:2 showed a value of 0.72 ± 0.08 kPa, not statistically significant compared to VG-3D. Interestingly, in this case, the higher viscosity of pre-crosslinked VG-based hydrogels reflected the higher modulus after the addition of ions.

Our results are far from the compressive modulus of the cartilage, because our hydrogel is thought to safely embed stem cells and let them differentiate into cartilage-like tissue over time (after a minimum of 28 days). For this reason, the mechanical properties of VG-based hydrogel do not have to be comparable with those of the cartilage. In fact, the mechanical properties of the cartilage are different, based on the zone considered: the mechanical properties of superficial and deep cartilage in terms of Young’s modulus are 280 ± 160 and 730 ± 260 kPa, respectively [50]. The goal of this work is to obtain a one-zone hydrogel that is able to host stem cells and keep them viable for a cartilage regeneration strategy, which aims to recreate the ECM typical of the cartilage by using stem cells in the long-term. However, to achieve the mechanical properties of cartilage tissue, the strategy must be changed to obtain a complete substitution of the tissue with a biocompatible construct. For example, this type of construct can also be designed using synthetic materials (i.e., PEGDA) to improve the stiffness and the toughness of the substitute, but that may severely compromise the viability and differentiation potential of the stem cells in the long-term. We have followed this strategy in our previous work [45].

#### 3.2.3. Protein Diffusivity Analysis

Confocal images on cross-sectioned VG-3D and VG-RGD crosslinked hydrogels at both concentrations (1:1 and 1:2) are reported in Figure 2d. The unmarked controls (CTR-3D for VG-3D, CTR-RGD for VG-RGD) hydrogels did not show any relevant autofluorescence signal when not incubated with FITC-conjugated albumin. Results show that all hydrogel formulations let the protein diffusion all along with their thickness (detected by red dashed lines) in the incubation period. In fact, both VG-based hydrogels (for both 1:1 and 1:2 dilutions) revealed the protein diffusivity in the whole 3D constructs, suggesting their suitability for being embedded with cells [23,37]. It is well known that the rheological and mechanical properties of the hydrogels, as well as the nutrients diffusivity, represent crucial cues on the cell behavior by affecting cell proliferation and differentiation [37,51].

### 3.3. hASCs Characterization

Human ASCs antigenic characteristics and differentiation potential were proven, according to internationally accepted criteria for defining MSCs [47]. The antigenic profile of expanded hASCs at passages 3 and 4 confirmed that cells were more than 99% positive for CD73, CD90, CD105, and CD166, and negative for CD31, CD34, and CD45 (Figure S1a). By using a specific differentiating medium, hASCs proved osteogenic and chondrogenic differentiation capabilities (Figure S1b,c).

#### 3.3.1. Biological Evaluations of hASCs-Laden Hydrogels

**Figure 3:**
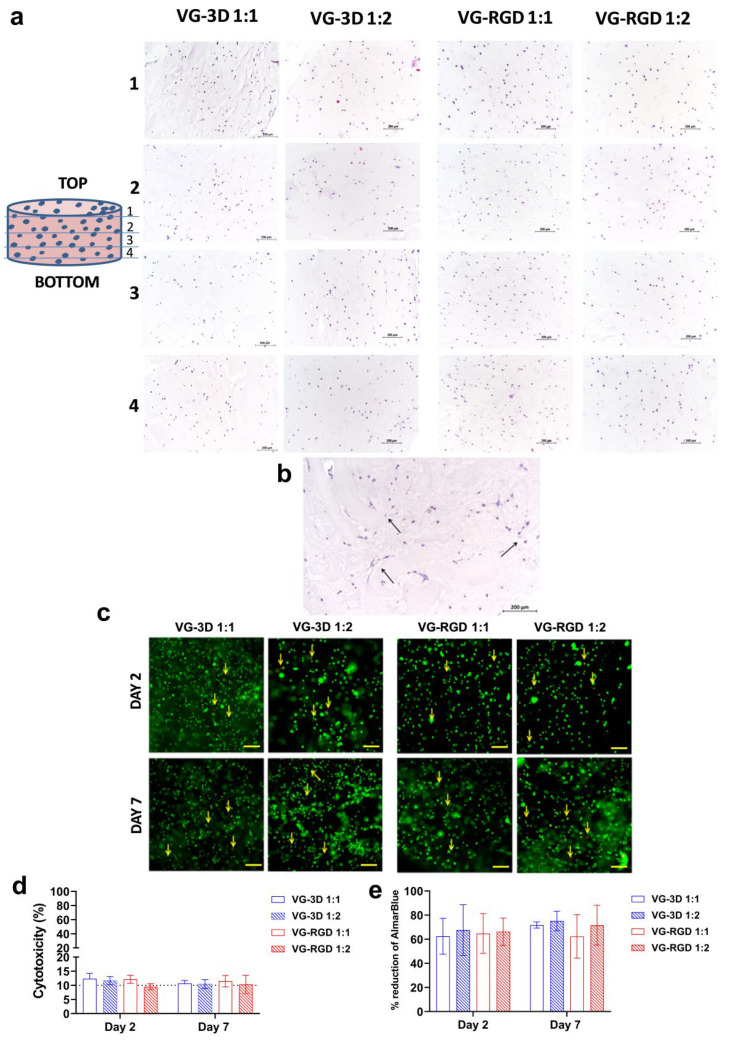
Histological analysis (hematoxylin-eosin staining): (**a**) Representative images of encapsulated-hASCs in VG-3D and VG-RGD hydrogels at day 7, scale bars = 200 µm. (**b**) Higher magnification of VG-RGD hydrogel 1:2, black arrows show hASCs cells. (**c**) Live/dead assay performed on hASCs encapsulated in VG-3D and VG-RGD hydrogels at days 2 and 7. Viable cells are shown in green; dead cells are shown in red (and indicated by yellow arrows); scale bars = 100 µm. (**d**) LDH analysis of hASCs embedded in VG-3D and VG-RGD hydrogels at days 2 and 7. Data are expressed as percentages of cytotoxicity. (**e**) AlamarBlue analysis of hASCs embedded in VG-3D and VG-RGD hydrogels at days 2 and 7. Data are expressed as percentage of reduction of alamarBlue. Data presented as mean ± SD, n = 5; *p*-values are calculated by Kruskal–Wallis with Dunn’s multiple comparisons test, as indicated in the statistical section.

Different hASCs characteristics in terms of morphology, distribution, viability, cytotoxicity, and metabolic activity were analyzed at days 2 and 7 after their encapsulation in the hydrogels. As shown in Figure 3a, we found that the cells were homogeneously distributed in all layers analyzed in VG-3D and VG-RGD hydrogels at both concentrations tested (1:1 and 1:2), suggesting a uniform material structure that promoted cell adhesion. These results confirmed the suitability of the hydrogel systems for the long-term maintenance of cells. After 7 days of culture, we observed a morphological change of hASCs in VG-RGD with respect to VG-3D hydrogels (Figure 3b). The cells displayed a fibroblast-like morphology in VG-RGD, while in VG-3D, they showed a more rounded shape, independent from the hydrogel concentration. A reproducible specific dimension (diameter: 10 mm, height: 2 mm) of the hydrogels was selected to assure a good comparison among the biological tests and good diffusion of the nutrients, as we showed in Figure 2d. Moreover, preliminary experiments were performed to select the hASCs density that better showed a uniform distribution of the cells inside the hydrogels, with defined dimensions and the best chondrogenic differentiation (data not shown).

These pre-defined conditions showed that hASCs showed a high percentage of viable cells (Figure 2b) until day 7, confirming that the chosen hydrogels size did not negatively affect the cell viability. Live/dead staining showed a high number of viable cells in both VG-3D and VG-RGD hydrogels (1:1 and 1:2) from day 2 to day 7 (Figure 3c). This observation was confirmed by the low number of dead cells (red) found in all hydrogels.

A lactate dehydrogenase (LDH) assay was used to assess the hydrogel cytotoxicity on hASCs, and we found that the detected enzyme was lower or equal to 10% in all hydrogels (Figure 3d), supporting the non-toxic nature of the hydrogel matrices. Both formulations, with and without the RGD motifs, confirmed that the microenvironment created by the hydrogels did not negatively affect the encapsulated hASCs.

Moreover, the analysis of metabolic activity (Figure 3e) showed that at day 2 and 7, the number of metabolically active cells in the VG-RGD hydrogel (1:1 and 1:2) was similar to that found in the VG-3D hydrogels.

These results indicated that all hydrogel properties (both mechanical and biochemical) were favorable to maintain positive hASCs characteristics. Our results are in line with previous studies [52,53], showing different cell types (like immune, epithelial, and stem cells) encapsulated in VG-based hydrogels. The results demonstrated that these hydrogels were interesting matrices able to create a cell-friendly environment. In fact, the cell viability and phenotype were not affected in these in vitro 3D models. Results revealing the optimal cell viability and low cytotoxicity of hASCs encapsulated in both VG hydrogels represented an important pre-requisite that encouraged further tests focused on chondrogenic differentiation.

#### 3.3.2. RGD-Based Hydrogels Favor the Chondrogenic Processes

**Figure 4:**
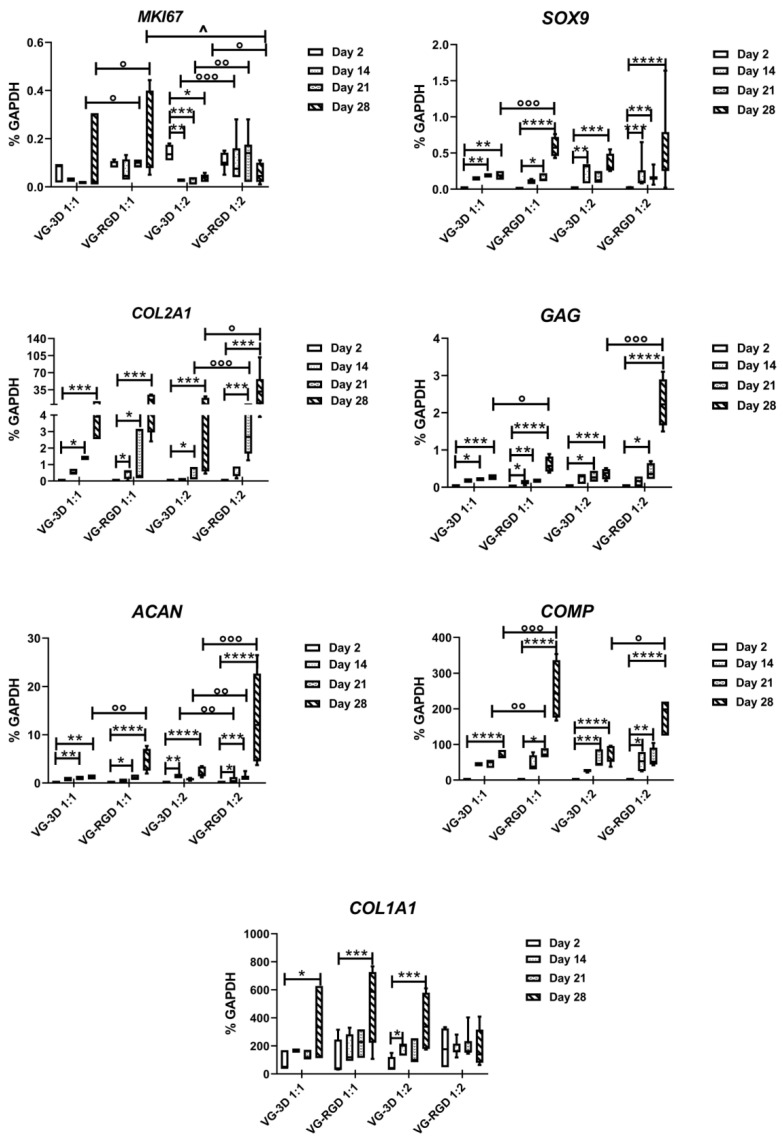
Real-time PCR analysis of M*Ki67, SOX9, COL2A1, GAG, ACAN, COMP,* and *COL1A1* genes of VG-3D and VG-RGD at day 2, 14, 21, and 28. Data were expressed as % *GAPDH* (housekeeping gene) and represented as a box-plot showing the median, minimum, and maximum values. The Kruskal–Wallis with Dunn’s multiple comparisons test was used for statistical analysis: * indicates differences along time points (day 2 to day 28) for each hydrogel (VG-3D and VG-RGD), ° indicates significant differences between different hydrogels (VG-3D versus VG-RGD) at the same concentration (1:1 or 1:2); ^ indicates differences between the same hydrogel at two different concentrations; n = 5, *, or ° or ^ *p* < 0.05, ** or °° *p* < 0.005, *** or °°° *p* < 0.0005, **** *p* < 0.0001.

The chondrogenic commitment of hASCs was analyzed at different time points (2, 14, 21, and 28 days) to check both the role of RGD motif and of the hydrogel concentrations (Figure 4). The analysis of the proliferating gene M*KI67* showed a significant decrease (indicated by *) from 2 to 28 days only in VG-3D 1:2, while no modulation was found in the other hydrogels analyzed. By contrast, we found a significant increase in M*KI67* in VG-RGD 1:1 compared to VG-3D 1:1 from day 21 to day 28, while a significant increase (indicated by °) was found in VG-RGD 1:2 compared to VG-3D 1:2 from day 14 to day 21. Interestingly, on day 28, the M*KI67* gene showed a significant decrease (indicated by ^) in VG-RGD 1:2 compared to 1:1.

The expression of the *SOX9* transcription factor gene showed an increase (indicated by *) from 2 to day 28 for all the hydrogels analyzed. On day 28, we also found a significant increase (indicated by °) in VG-RGD 1:1 compared to VG-3D 1:1. The analysis of typical chondrogenic *COL2A1, GAG,* and *ACAN* genes showed a significant increase (indicated by *) starting from day 14 or 21 until day 28 in all the hydrogels tested. However, of these three genes compared to the other formulations, only VG-RGD 1:2 showed the highest expression (indicated by °) on day 28. Interestingly, the expression of these three genes in VG-RGD 1:2 was also significantly higher (indicated by °) than in VG-3D 1:2. The *COMP* gene also significantly increased (indicated by *) from day 2 until day 28 in all formulations tested; however, on day 28, we saw a significant increase (indicated by °) in both VG-RGD 1:1 and 1:2 compared to VG-3D 1:1 and 1:2. The fibrotic *COL1A1* gene showed a significant increase (indicated by *) from day 2 to day 28 for all hydrogels tested, except for VG-RGD 1:2.

The analysis of the proliferating gene *MKI67* found that the presence of RGD motifs in the hydrogel favored cell proliferation; however, the 1:2 formulation was significantly decreased on day 28 compared to the 1:1 formulation, suggesting that this formulation better counteracted cell proliferation compared to the other conditions and better induced cell differentiation. In fact, we demonstrated that the VG-RGD 1:2 formulation showed the highest expression of *COL2A1*, *GAG*, and *ACAN,* the three major chondrogenic genes, on day 28, indicating that the presence of the RGD motif positively influenced the chondrogenic commitment of the hASCs. In the ECM of the articular cartilage, we distinguish two supramolecular compartments, the fibrillar collagen network, with the main collagen type 2, and the extrafibrillar matrix network, with proteoglycan and aggrecan [54]. The two networks are interconnected by non-collagenous perifibrillar adapter matrix protein-like COMP [55,56]. The positive increase in the VG-RGD 1:2 formulation of different ECM genes that provide different functions to the cartilage, such as tensile strength and swelling pressure, clearly showed that this formulation facilitates the formation of a very close cartilage-like tissue. Interestingly, not only the presence of the RGD motif, but also the lower concentration (1:2) of the hydrogel positively contributed to the chondrogenic differentiation of hASCs, suggesting that both culture conditions and hydrogel microenvironment at a defined stiffness were important for providing a positive in vitro effect on cell differentiation and ECM production.

**Figure 5:**
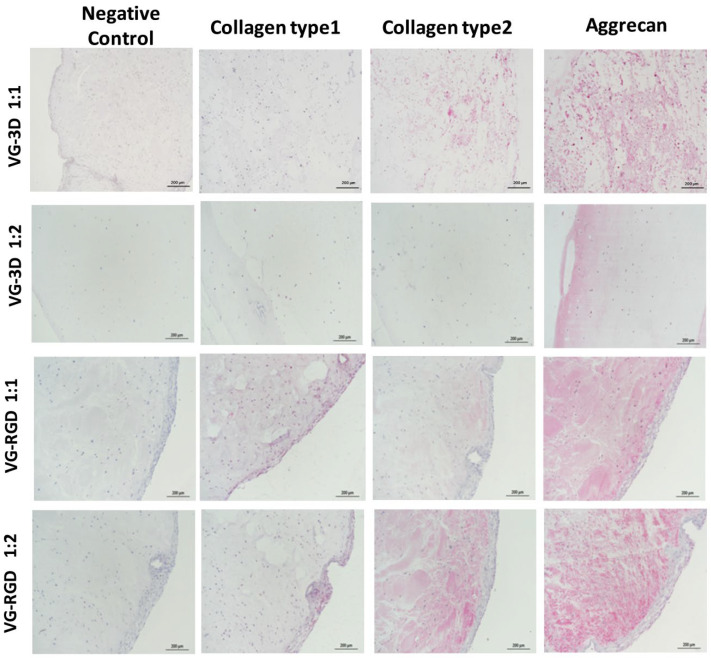
Immunohistochemical analysis of collagen type 1, collagen type 2, and aggrecan on encapsulated hASCs in VG-3D and VG-RGD hydrogels (1:1 and 1:2) at day 28. The negative control was included. Pink areas = positive immunostaining; scale bars = 200 µm.

The different effect of RGD motif on hASCs chondrogenesis was then investigated at day 28 by analyzing the modulation of collagen type 1, collagen type 2, and aggrecan proteins, which are typical fibrotic and chondrogenic markers (Figure 5). It was observed that chondrogenic treatment associated with VG-RGD 1:2 caused, in each sample analyzed, higher positive staining to both aggrecan and collagen type 2 with respect to VG-3D 1:1 and 1:2 (Figure 5). The presence of positive areas, mainly in the VG-RGD 1:2, confirmed that this formulation and not the other which also tested positively, pushed the differentiation of the hASCs. It has been shown that the concentration of the -RGD motif in the hydrogels could be detrimental to or supportive of cell differentiation [38,39,40,43]. In our in vitro system, we demonstrated that the presence of the RGD motif in the hydrogel significantly contributed to the commitment of hASCs, as reported by other authors using different hydrogel types [40,41,44]. It has been suggested that defined RGD concentration [57] and nanoscale spatial distribution [58] associated with appropriate mechanical stimulation [59] are effective at inducing the chondrogenic phenotype of the cells.

Conversely, chondrogenic differentiated areas were almost completely nonresponsive to collagen type 1. Therefore, despite the heterogeneity of the cell population, the embedded hASCs showed a very similar trend of response to chondrogenic commitment. A time response of RGD to cell differentiation [60,61] has been shown, contributing to the confirmation of the important role of this tri-peptide in inducing chondrogenic differentiation.

## 4. Conclusions

In the field of cartilage tissue engineering, an ideal injectable hydrogel should be able to reproduce the complex functionalities of the cartilaginous ECM, thus providing specific cell adhesion and the subsequent control of cellular functions directing new tissue formation. Natural polysaccharides from different sources are potentially exciting candidates for cartilage regeneration. The RGD motif represents a crucial component of the adhesive proteins in the ECM, and via the α_v_β3 integrin receptors, favor cellular attachment that leads to the activation of focal adhesion kinase (FAK), well known to transmit the adhesion-dependent cell survival signal within the cell [41]. This study demonstrates that VG-RGD hydrogels at the concentration of 1:2 significantly contribute to guaranteeing the chondrogenic commitment of the hASCs laden in the hydrogel. Both the rheological and biochemical characteristics of this hydrogel formulation, with respect to other counterparts, positively influenced hASCs chondrogenic differentiation. Future studies may focus on the role of the hydrogel formulations in a broader panel of integrin markers of hASCs to define which is the integrin signaling directly involved in the control of the hASCs chondrogenic differentiation, as previously reported for specific integrins [41,62].

## Figures and Tables

**Table 1:** Rheological indexes (**n**, **K**) extracted by flow curves of VG-3D and VG-RGD for dilution 1:1 and 1:2, and used to calculate shear stresses on cells during injection with 26 G (inner diameter: 0.25 mm) needles. Data are reported as mean ± SD, n = 5.

	VG-3D 1:1	VG-3D 1:2	VG-RGD 1:1	VG-RGD 1:2
**K [Pa·s^n^]**	3.36 ± 0.69	1.56 ± 0.52	2.03 ± 0.38	1.12 ± 0.36
**n**	0.21 ± 0.02	0.22 ± 0.09	0.20 ± 0.03	0.29 ± 0.04
**τ [Pa]**	27.12 ± 9.01	15.38 ± 8.24	21.33 ± 8.48	15.93 ± 5.12

## Data Availability

The data presented in this study are available on request from the corresponding author. The data are not publicly available due to issues of privacy.

## Supplementary Materials

The following supporting information can be downloaded at: https://www.mdpi.com/article/10.3390/gels8060382/s1, Figure S1: hASCs characterization; Table S1: Oligonucleotide primers used for real-time PCR.
