# Supplementary material for: RGD-Functionalized Hydrogel Supports the Chondrogenic Commitment of Adipose Mesenchymal Stromal Cells
Source: Gels. 2022 Jun 15;8(6):382. doi: 10.3390/gels8060382 (PMC9222613; doi:10.3390/gels8060382)
Supplement: Supplementary file 1 [file gels-08-00382-s001.zip › gels-1761470-supplementary.pdf]

Figure S1: hASCs characterization

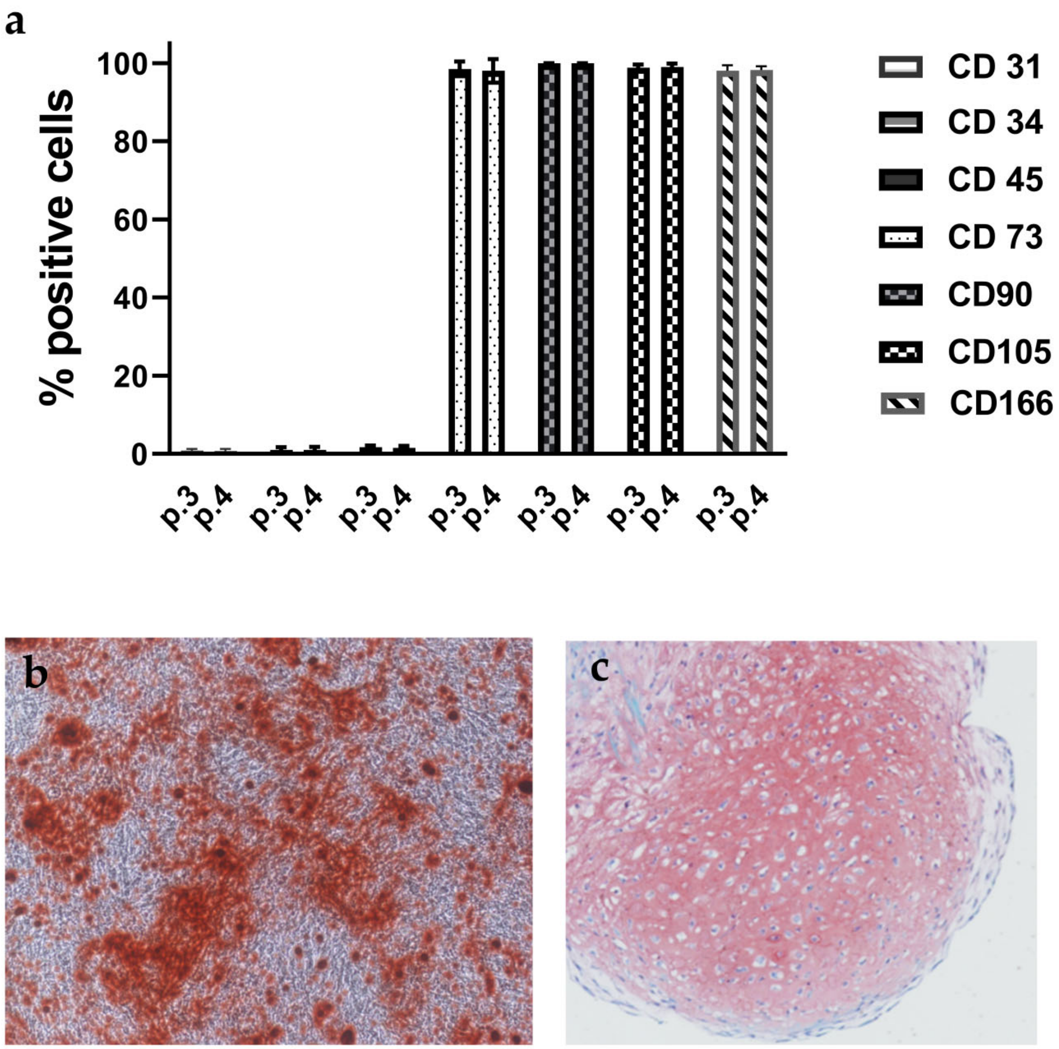

a) Antigenic Characaterization of hASCs at both passage 3 (p.3) and passage 4 (p.4), data are expressed as % of positive cells (mean ±SD). b) Alizarin Red staining on hASC ostegenic differentiated at day 21. c) Safranin O staining on hASC chondrogenic differentiated at day 28.

Table S1: Oligonucleotide primers used for real-time PCR**.**

Target Gene	Primers (forward and reverse)	Product size (bp)	GenBank Accession No.	Primer Efficiency (%)
ACAN	TCGAGGACAGCGAGGCC<br>TCGAGGGTGTAGCGTGTAGAGA	85	NM_001135	96,5
COL1A1	CCTGGATGCCATCAAAGTCT<br>CGCCATACTCGAACTGGAAT	170	NM_000088	95,4
COL2A1	GACAATCTGGCTCCCAAC<br>ACAGTCTTGCCCCACTTAC	228	NM_001844	98,1
COMP	Biorad unique assay ID:<br>qHsaCID0021064	148	NC_000019.9<br>NG_007070.1<br>NT_011295.11	95
GAG	Biorad unique assay ID:<br>qHsaCID0005989CSPG4	148	NC_000015.9<br>NT_010194.17	101
GAPDH	CGGAGTCAACGGATTTGG<br>CCTGGAAGATGGTGATGG	218	NM_002046	101,9
MKI67	TCTGGTAATGCACACTCCACC<br>GCTTTGTGCCTTCACTTCCAC	112	NM_002417	98,1
SOX9	GAGCAGACGCACATCTC<br>CCTGGGATTGCCCCGA	281	NM_000346	97,2
